# Combined nanopore adaptive sequencing and enzyme-based host depletion efficiently enriched microbial sequences and identified missing respiratory pathogens

**DOI:** 10.1186/s12864-021-08023-0

**Published:** 2021-10-09

**Authors:** Mingyu Gan, Bingbing Wu, Gangfeng Yan, Gang Li, Li Sun, Guoping Lu, Wenhao Zhou

**Affiliations:** 1 Center for Molecular Medicine, Pediatric Research Institute, Children’s Hospital of Fudan University, National Children’s Medical Center, Shanghai, China; 2 Department of Pediatric Emergency and Critical Care Medicine, Children’s Hospital of Fudan University, National Children’s Medical Center, 399 Wanyuan Road, Shanghai, 201102 China; 3 Department of Rheumatology, Children’s Hospital of Fudan University, National Children’s Medical Center, Shanghai, China; 4 Department of Neonates, Key Laboratory of Neonatal Diseases, Ministry of Health, Children’s Hospital of Fudan University, National Children’s Medical Center, Shanghai, China

**Keywords:** Metagenomics, Nanopore adaptive sequencing, Host depletion, Microbe enrichment

## Abstract

**Background:**

Enzyme-based host depletion significantly improves the sensitivity of clinical metagenomics. Recent studies found that real-time adaptive sequencing of DNA molecules was achieved using a nanopore sequencing machine, which enabled effective enrichment of microbial sequences. However, few studies have compared the enzyme-based host depletion and nanopore adaptive sequencing for microbial enrichment efficiency.

**Results:**

To compare the host depletion and microbial enrichment efficiency of enzyme-based and adaptive sequencing methods, the present study collected clinical samples from eight children with respiratory tract infections. The same respiratory samples were subjected to standard methods, adaptive sequencing methods, enzyme-based host depletion methods, and the combination of adaptive sequencing and enzyme-based host depletion methods. We compared the host depletion efficiency, microbial enrichment efficiency, and pathogenic microorganisms detected between the four methods. We found that adaptive sequencing, enzyme-based host depletion and the combined methods significantly enriched the microbial sequences and significantly increased the diversity of microorganisms (*p* value < 0.001 for each method compared to standard). The highest microbial enrichment efficiency was achieved using the combined method. Compared to the standard method, the combined method increased the microbial reads by a median of 113.41-fold (interquartile range 23.32–327.72, maximum 1812), and the number of genera by a median of 70-fold (interquartile range 56.75–86.75, maximum 164). The combined method detected 6 pathogens in 4 samples with a median read of 547, compared to 5 pathogens in 4 samples with a median read of 4 using the standard method.

**Conclusion:**

The combined method is an effective, easy-to-run method for enriching microbial sequences in clinical metagenomics from sputum and bronchoalveolar lavage fluid samples and may improve the sensitivity of clinical metagenomics for other host-derived clinical samples.

**Supplementary Information:**

The online version contains supplementary material available at 10.1186/s12864-021-08023-0.

## Background

Infectious diseases remain the major threat to human health, especially for children under the age of 5 [1]. Rapid and accurate detection of the causative agent is the key to treatment [2]. With the development of sequencing technology, metagenomics sequencing has been widely used for clinical pathogen detection [3–5]. Metagenomic sequencing is able to sequence all nucleic acids in samples and detect all potential pathogens, even emerging pathogens [6–8].

However, a high background of host DNA in clinical samples impedes the detection of pathogens [9]. This shortage of clinical metagenomics may be overcome via microbe enrichment [3, 9–11]. 16S rDNA sequencing is very effective in profiling microbial diversity [12–14]. However, 16S sequencing for clinical diagnosis misses important viral and fungal pathogens in the respiratory tract. The direct enrichment of viruses using spiked primers achieved a median of tenfold enrichment [15]. However, this type of method only enriches limited number of microbes, which nullifies the major advantage of metagenomic sequencing. Experimental host DNA depletion methods enable relative microbe enrichment [3, 16, 17]. Charalampous et al. used the saponin-based differential lysis method to deplete host DNA, which resulted in a maximum 10^4^-fold depletion of host DNA and maximum 100-fold enrichment of microbe DNA [3].

Nanopore sequencing is characterized by long read sequencing and real-time data analysis, which is important for the rapid identification of pathogens and suspected emerging pathogens [3, 18, 19]. Nanopore sequencing allows computationally targeted sequencing, which is known as Read Until mode [20]. Signals in this mode were analyzed in real-time after a DNA molecule entered a pore. The beginning of the sequence strand was rapidly mapped to the provided reference sequence. If the sequence was located in the targeted region, or was not a sequence to be depleted, the DNA molecule was allowed to continue sequencing. If the sequence was not the targeted sequence, or was to be depleted, the DNA molecule was ejected from the pore. Based on this function, Alexander et al. developed a toolkit (readfish) and showed a 5.7-fold increase in a relatively low abundance microbe in the ZymoBIOMICS mock metagenomic community [21]. Another study developed UNCALLED software, which uses raw signals to compare with the reference sequence. They showed a 4.46-fold enrichment of yeast sequence [22].

However, few studies applied nanopore adaptive sequencing to clinical samples for microbe enrichment. The difference in enrichment efficiency between enzyme-based host depletion and nanopore adaptive sequencing is not known. Alternatively, the best approach may be to combine these two methods to efficiently enrich microbes. The present study hypothesized that the combination of experimental host depletion and adaptive sequencing would produce the best microbe enrichment efficiency compared to the use of either method alone. Four groups of results for each clinical sample were acquired by combining standard DNA extraction and enzyme-based host depletion with standard and adaptive sequencing. We showed a median of 113.41-fold microbe enrichment using the combination method of enzyme-based host depletion and adaptive sequencing.

## Results

### Study design

To evaluate the microbe enrichment efficiency of experimental enzyme-based host depletion, nanopore adaptive sequencing and the combination of the two methods, we designed the experimental procedure illustrated in Fig. S1. At first, each sample was divided into two parts with the same volume. Each part was processed using standard DNA extraction and enzyme-based host DNA depletion respectively. For each DNA, standard sequencing and adaptive sequencing were performed simultaneously on the same flow cell. Therefore, each sample was processed using four methods: standard DNA extraction with standard sequencing (SD_SSD, the standard), standard DNA extraction with adaptive sequencing (SD_ADS, using adaptive sequencing alone), host depletion with standard sequencing (HD_SSD, using enzyme-base host depletion alone), and host depletion with adaptive sequencing (HD_ADS, the combined method). We collected 8 sputum or bronchoalveolar lavage fluid (BALF) samples from children with pneumonia or pulmonary infection (Table S1).

### Nanopore sequencing results

Nanopore sequencing was performed on the ONT GridION platform. Because samples were sequenced on different flow cells with various numbers of active pores, the reads and bases generated were not equal between samples (Table S2). For each sample, adaptive sequencing and standard sequencing were performed on the same flow cell by setting the adaptive sequencing channel from 1 to 256. Therefore, we compared the sequencing outputs between adaptive sequencing and standard sequencing. There was no consistent trend between the number of reads yielded by adaptive and standard sequencing. However, except for the enzyme host depleted sample of P1, adaptive sequencing yielded fewer bases than standard sequencing in all of the other samples. This result may be caused by the shorter sequence length of adaptive sequencing (Table S2 and Fig. S2).

### Adaptive sequencing reads

The Oxford nanopore sequencer enables selective sequencing by controlling the driving voltage across individual nanopores. Therefore, three types of reads are yielded from adaptive sequencing. According to Oxford nanopore’s definition, “unblock” reads represent the DNA molecules rejected by the pore, and the first few hundred bp of these DNA molecules are sequenced. The “stop receiving” reads mean DNA molecules accepted by the pore, and the full length of the molecules should be sequenced. The “no decision” reads indicate that DNA molecules cannot be decide to be rejected or accepted. We compared the percentage of the three types of reads. We observed “stop receiving” reads in the SD_ADS group (Fig. S3A), but few in the HD_ADS group (Fig. S3B). The percentage of “stop receiving” reads in the SD_ADS group was lower than the “unblock” and “no decision” reads in each sample. However, the long length of “stop receiving” reads resulted in an increased percentage of bases (Fig. S3A, Fig. S4A). For the “unblock” and “no decision” reads, we found that except for the P4 and P6 samples, the “unblock” reads and bases were higher than the “no decision” reads and bases in the HD_ADS group (Fig. S3B). However, we didn’t observe the same trend in SD_ADS (Fig. S3A). The length of the “unblock” reads was smaller than the “no decision” reads in both groups, and the length of the “unblock” reads was smaller than the “stop receiving” reads (Fig. S4). The “stop receiving” reads indicate the accepted reads, which means the microbial reads in our study. However, we found that on average 93.42% of the “stop receiving” reads could be mapped to the human genome.

### Human sequence depletion

The enzyme-based host depletion (HD) method resulted in shorter reads than standard DNA extracted (SD) method (Fig. S2). Most of the long reads of the SD group were classified as human (e.g. greater than 99% of the reads longer than 5000 bp belonged to humans), which indicates that long human reads were depleted by the HD method. Adaptive sequencing (ADS) also significantly depleted long human reads (Fig. S2).

**Fig. 1:**
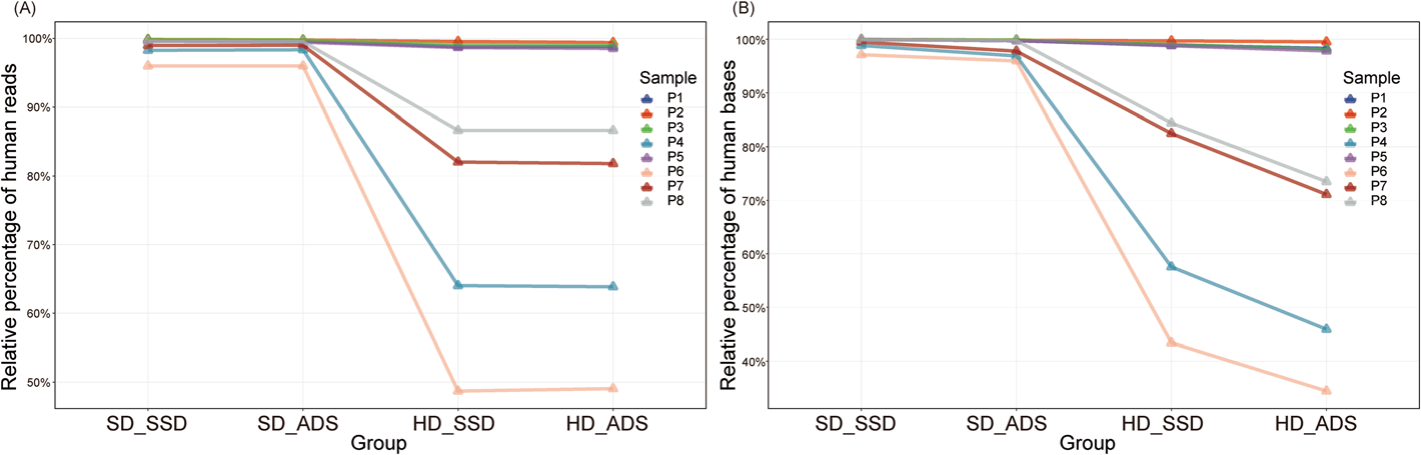
Human sequence depletion. Relative abundance of (**A**) host reads, and (**B**) host bases

Reads were classified into four classes, including human, *E. coli*, unclassified and microbe reads (Table S3). We observed that a large part of the nonhuman reads in the SD_SSD and SD_ADS groups were classified as *E. coli* (Table S3). However, most of these reads only mapped to a specific region in the *E. coli* genome (Fig. S5), which suggests reagent contamination in the Ligation Sequencing Kit (SQK-LSK109). After excluding unclassified and *E. coli* reads, the relative abundances of human and microbe reads in each sequencing sample were calculated. A significant decrease in human reads was found in four samples (P4, P6, P7 and P8) but only between the SD and HD groups (Fig. 1, Table S3). For human bases, a continuous significant decrease in human bases was found in four samples (P4, P6, P7 and P8), following the order of SD_SSD, SD_ADS, HD_SSD and HD_ADS (Fig. 1, Table S3). The reduction in bases indicates that in addition to the HD method, the ADS method was also effective in removing human sequences. The different reduction patterns between human reads and bases occurred because the ADS method requires the identification of a short sequence to determine whether it is human. This step results in the output of short human reads (i.e., the “unblock” reads illustrated in Fig. S3 and Fig. S4).

### Microbial sequence enrichment

**Fig. 2:**
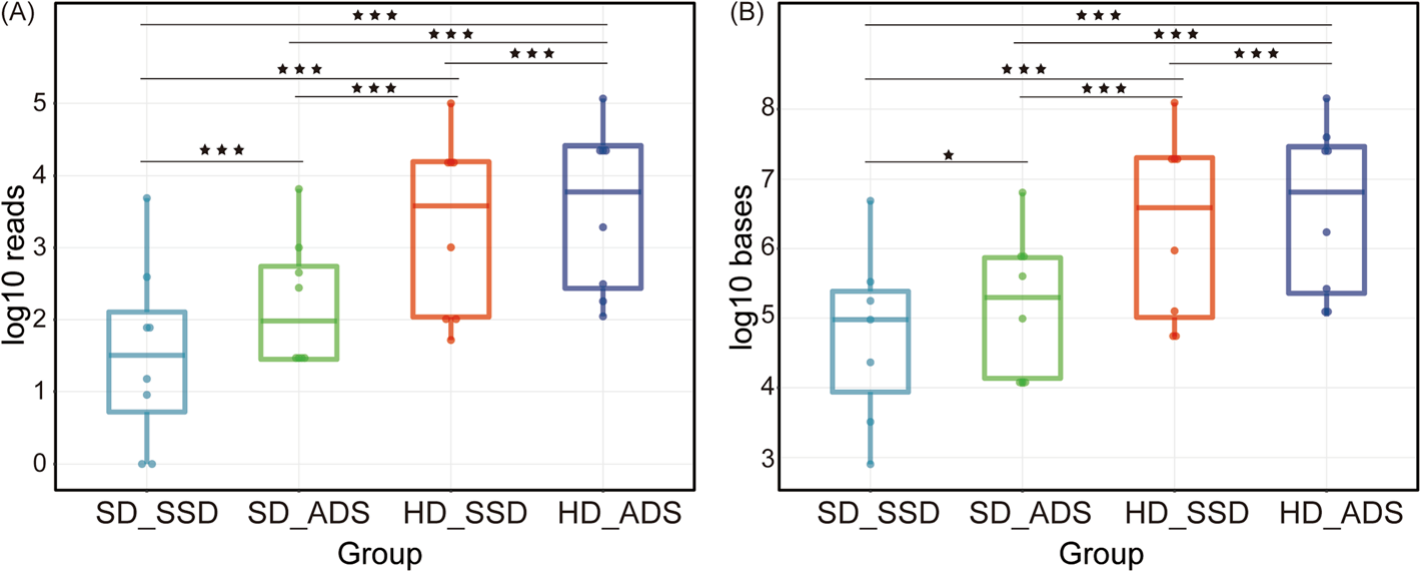
Microbial sequence enrichment. **A** Microbial reads and (**B**) microbial bases detected by the four methods. SD_SSD: the standard method, SD_ADS: using adaptive sequencing alone, HD_SSD: using enzyme-base host depletion alone, HD_ADS: the combined method. Significant difference was found between each combination among the four methods

The respiratory tract contains a wide variety of colonizing microorganisms. After identifying the microbial species of the sequenced reads, we summed the reads of all microorganisms. We compared the normalized number of reads and bases of microorganisms obtained using the four methods. We found that the HD and ADS methods alone and the combined HD and ADS methods were significantly enriched for microorganisms (Fig. 2, Table S4). The results of all four methods were significantly different from each other (*p* value < 0.001). The HD method alone enriched more microorganisms than the ADS method alone. The combination of the HD and ADS methods enriched most microorganisms. Compared to the standard method, ADS alone enriched microbial reads by a median of 3.59-fold (interquartile range 2.39–10.34, maximum 33), HD alone by a median of 62.96-fold (interquartile range 18.18–144.12, maximum 1072), and the combined method HD_ADS by a median of 113.41-fold (interquartile range 23.32–327.72, maximum 1812).

### Increased microbial diversity

**Fig. 3:**
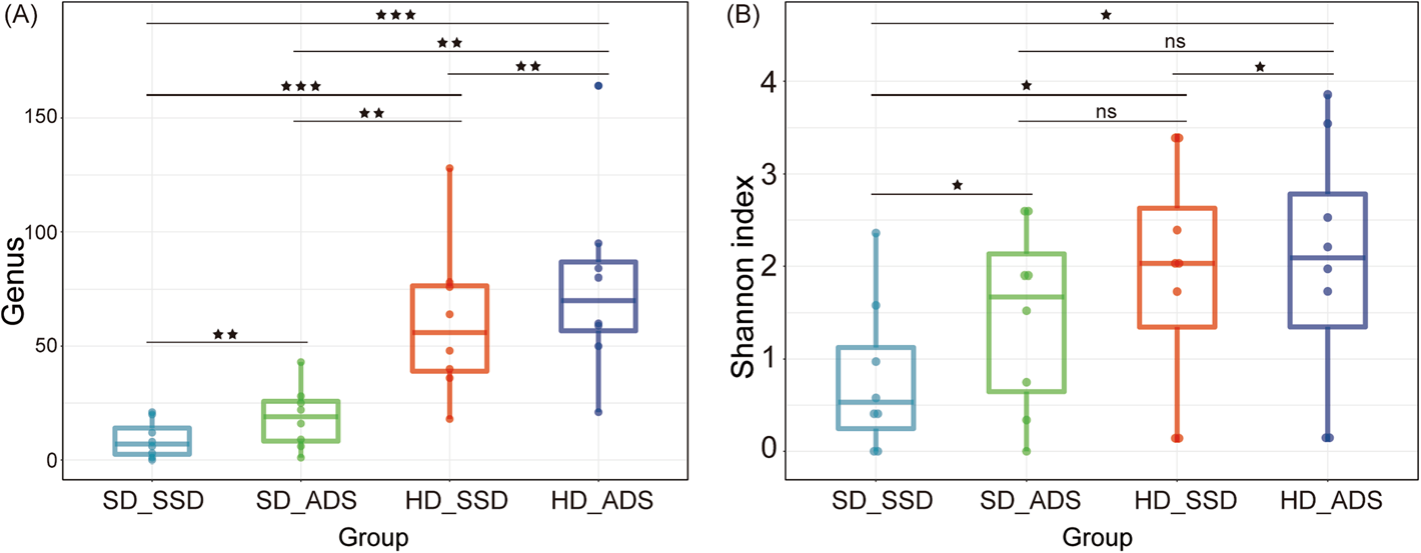
Microbial diversity. **A** Genus detected by the four method. **B** Alpha diversity of the four methods

We also compared the number of genera and alpha diversity of the microorganisms detected using the four methods (Fig. 3). We found that, similar to the microbial sequence enrichment results, the HD and ADS methods alone and the combined method significantly increased the number of genera and alpha diversity. The number of microbial genera and alpha diversity increased more with the HD method alone than with the ADS method alone. The combined method had the highest enrichment efficiency for microbial genera (median 70-fold, interquartile range 56.75–86.75, maximum 164). However, there was no significant difference in alpha diversity between the combined method and the HD method alone, ADS method alone and HD method alone.

**Fig. 4:**
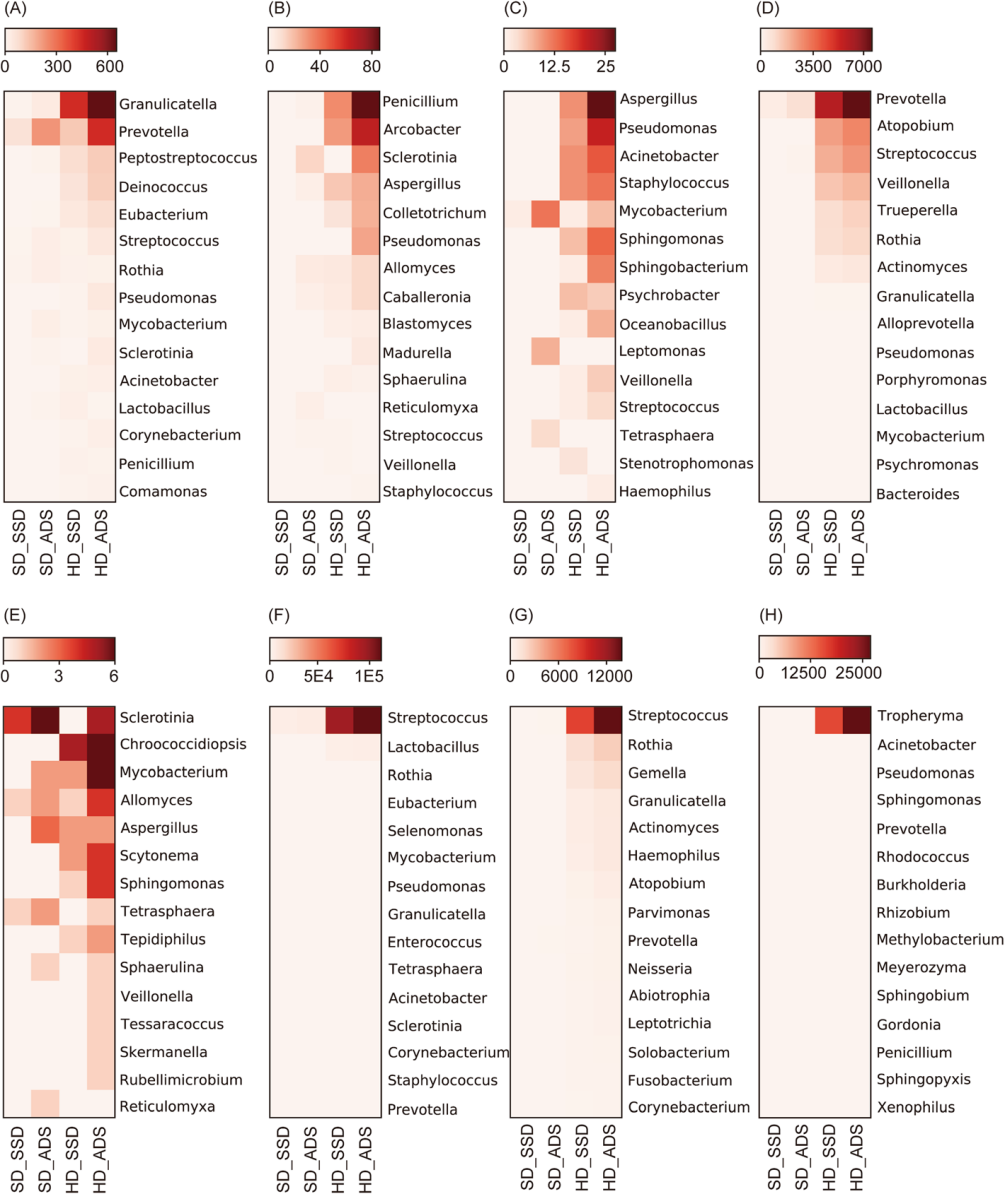
Top 15 microbial genus detected by the four methods in each sample. **A**-**H** indicates P1-P8 patients

The top 15 microorganisms in each sample are shown in Fig. 4. Except the increase microbial diversity, we also found that the HD, ADS and the combined methods increased the sequencing reads for each microorganism (Fig. 4). For these top 15 microorganisms, ADS method increases the reads by a median of 2.83-fold (interquartile range 2.34–4.16, maximum 19), HD method increases the reads by a median of 37.17-fold (interquartile range 15.31–163.15, maximum 1232), the combined method increases the reads by a median of 47.14-fold (interquartile range 21.56–278.75, maximum 2081).

### Pathogenic microorganisms detected

The aim of enriching microorganisms by host depletion is to increase the detection sensitivity of pathogenic microorganisms. Therefore, we compared the pathogenic microorganisms detected using the four methods. We invited experienced laboratory experts to identify pathogenic microorganisms from microbial classification results by reviewing medical records. Possible pathogens were identified in 4 patients (Table 1). For the other 4 patients, we did not detect any pathogenic microorganisms in the standardized results (Table S4) or the raw results (Table S5). Patient P4’s chest X-ray suggested pneumonia and severe lung infection. *S. pneumoniae* was detected by all four methods, and the number of reads increased progressively in the order of SD_SSD, SD_ADS, HD_SSD and HD_ADS. The child had a pulmonary infection and was given cefotaxime. However, the child suffered from acute liver failure and was in critical condition. The family requested to abandon the treatment and the child was discharged automatically. Patient P6 had recurrent fever, cough, shortness of breath, coarse breath sounds in both lungs, and pulmonary exudate as suggested by CT. *S. pneumoniae* was detected by all four methods, and the number of reads increased progressively in the order of SD_SSD, SD_ADS, HD_SSD and HD_ADS. The child was diagnosed with severe combined immunodeficiency disease (IL2RG gene mutation) and was given meropenem, fosfomycin, voriconazole, caspofungin, teicoplanin, and sulfofen for anti-infection. However, the child was heavily infected and had multiple organ insufficiency. After aggressive anti-infective treatment, the child still had a fluctuating temperature, and no signs of improvement of multiple organ insufficiency. After careful consideration, the child’s family requested a discharge. Patient P7 had juvenile idiopathic arthritis, and a CT scan of the chest showed multiple ground glass shadows in both lungs. BALF was collected for further diagnosis of connective tissue disease-associated interstitial lung disease and lung infection. *S. pneumoniae* and *S. dysgalactiae* were detected by all four methods. *H. parainfluenzae* was detected by three methods except SD_SSD. *S. dysgalactiae* was also detected by culture. According to the culture results, anti-infection treatment with ampicillin and sulbactam was administered intravenously. The child was cured and discharged from the hospital. Patient P8 had childhood dermatomyositis and was admitted with a diagnosis of juvenile dermatomyositis involving the lungs. Lung CT showed localised interstitial changes in both lungs. With the concern of a specific pathogenic opportunistic infection, BALF was collected. Consistent with clinician’s concern, an extremely rare pathogen was detected. *T. whipplei* was detected by all four methods, and the number of reads detected had the same trend as the rest of the patients. The child was treated with ceftriaxone for 2 weeks in combination with oral anti-infective treatment with sulfamethoxazole. After treatment, the child was in good general condition and was discharged.

**Table 1:** Pathogens detected by nanopore sequencing and culture, stratified by four methods

Sample	Pathogen	SD_SSD		SD_ADS		HD_SSD		HD_ADS		Culture
P4	*S. pneumoniae*	4/2.0 k^a^	180.9 k/792.7 M^b^	16/16.0 k	915 k/792.7 M	39/43.6 k	950.5 k/792.7 M	65/71.4 k	1484 k/792.7 M	Negative
P6	*S. pneumoniae*	20/14.1 k	48.7 k/74 M	38/27.5 k	137.8 k/74 M	503/470.2 k	63.1 k/74 M	557/504.1 k	80.2 k/74 M	Negative
P7	*S. pneumoniae*	1/331	40.8 k/168 M	4/1.7 k	152.6 k/168 M	363/308.0 k	159.5 k/168 M	531/439.8 k	261 k/168 M	*S. dysgalactiae*
	*H. parainfluenzae*	0/0		7/15.0 k		333/323.8 k		538/535.2 k		
	*S. dysgalactiae*	1/4.0 k		8/10.1 k		1269/1.3 M		2102/2.1 M		
P8	*T. whipplei*	13/20.9 k	35.6 k/195.7 M	28/96.8 k	72 k/195.7 M	15,951/21.5 M	162 K/195.7 M	26,929/36.1 M	276.5 K/195.7 M	Negative

^a^Number of reads / Number of bases for the pathogen

^b^Total number of reads / Total number of bases for the sample

## Discussion

The extremely high proportion of host nucleic acids in clinical samples can drown out microbial sequences and has important implications for the sensitivity of clinical metagenomics [9, 10]. The present study compared the microbe enrichment efficiency of enzyme-based host depletion, adaptive sequencing, and the combination of these two methods. We found that all three methods significantly enriched the sequences of microorganisms. The sequencing read enrichment efficiency of the combined method was significantly higher than the other methods and reached a median of 113.41-fold. The three methods also significantly increased the diversity of microorganisms detected. The combined method had the highest enrichment efficiency for microbial genera (median 70-fold), which was significantly higher than the other methods. The results for pathogens detected followed the same trend, with the combined method achieving the highest positivity rate, the number of pathogenic microorganisms detected and the corresponding number of reads. The results of this study provide a new microbial enrichment strategy for clinical metagenomics.

Enrichment of microorganisms effectively increases the positive rate of clinical metagenomics [10, 15, 23]. Current enrichment methods are divided into two major groups: methods that directly enrich microorganisms and methods that deplete the hosts. 16S sequencing directly enriches the 16S rRNA of microorganisms and is widely used to study microbial communities in various human ecological niches. These niches include microbial-rich sites (i.e., the gastrointestinal tract, the lower respiratory tract) and microbial-rare sites (i.e., blood and womb, which are normally considered sterile) [12–14]. It demonstrates the strong enrichment ability of 16S sequencing. However, for clinical diagnosis, 16S sequencing is not as applicable as metagenomics sequencing because it cannot detect viruses and fungi. In addition, short-sequencing of 16S sub-regions does not fully satisfy microbial specie-level identification [24]. The spiked-primer-based method targets 14 viruses, and achieves a median of 10-fold enrichment [15]. However, the targets for microbial enrichment are generally limited, which loses the major advantage (untargeted detection) of metagenomics [15, 25, 26]. Host depletion methods include filter-based, CpG methylation-based, mammalian cell selective lysis, and propidium monoazide-based methods [11, 16]. Filter-based methods cannot deplete extracellular microbial DNA [16]. Methylation-based methods are not suitable for microorganisms with methylation patterns that are similar to eukaryotes. Selective lysis depletes host DNA by first lysing the host cell then degrading the released host DNA. Selective lysis is the most widely used method in clinical metagenomics. Real-time nanopore adaptive sequencing recently enabled the efficient enrichment of target sequences [21, 22]. This method compares sequencing reads to target sequences in real time, which enable real-time control of DNA molecules. Because the method achieves targeted enrichment during sequencing, the principle is completely different from the previous method, which makes it possible to use the two methods in combination. The results of this study also demonstrated that combining the two methods was more effective than using either method alone.

We observed a significant reduction in the relative proportions of host reads and bases in four samples (P4, P6, P7 and P8), but the remaining four samples showed no significant change. This result may be associated with the absolute content of microbial sequences in the samples. We observed that in the standard group without any methodological treatment (SD_SSD), significantly higher microbial sequences were detected in P4, P6, P7 and P8 than the other 4 samples, and the microbial content also correlated with the trend of host depletion. This result suggests that higher microbial content improves the host depletion effect for the clinical samples.

The present study used GRCh38 as the reference sequence to remove human sequences. However, the inclusion of partially inserted viral sequences in this reference genome may have resulted in poor enrichment efficiency for this class of viruses [27]. Although no sequences derived from this class of viruses were observed in this study, future studies should modify the reference sequence to obtain the corresponding viral sequence.

Two patients enrolled in this study had a clinical diagnosis of interstitial pneumonia with suspected viral infection. However, we did not detect the virus using any of the four methods. This result may be because that the viral capsid is more fragile than the cell wall of bacteria, which resulted in the degradation of viral DNA during selective lysis. Previous studies also suggested that selective lysis led to a bias towards Gram-positive bacteria [28]. Therefore, the limitations of this method must be considered when using a selective lysis method.

One limitation of this study is the limited sample size, which made it impossible to compare the positive detection rates of pathogenic microorganisms between the different methods. However, our results showed that the combined method significantly enriched the microorganisms. Another limitation is the lack of validation of the pathogenic microorganisms detected. The positive rate of culture was too low to validate the results of the metagenomics testing. However, with the exception of patient P8, the pathogenic microorganisms identified were all common respiratory pathogens, including hospital-acquired pathogenic microorganisms. Patient P8 has childhood dermatomyositis. He was treated with long-term oral high-dose hormone and immunosuppressive therapy, which may lead to opportunistic infections with specific pathogens. Because of the extremely low concentration of DNA after host depletion, the starting conditions for ligation sequencing library construction (SQK-LSK109) were not met. Therefore, we used a PCR-based library construction kit (SQK-PSK004). This resulted in different library construction procedure for the HD and SD samples. The effect of PCR on the results during the library constructing process cannot be excluded.

## Conclusions

The present study showed that the combination of enzyme-based host depletion and nanopore adaptive sequencing reached the highest microbial enrichment efficiency and positive detection rate of pathogenic microorganisms. This study provides a new strategy for microbial enrichment and improvement of the positive detection rate of pathogenic microorganisms.

## Methods

### Clinical sample collection

Patients in the Children’s Hospital of Fudan University with an admission diagnosis of pneumonia or pulmonary infection were prospectively enrolled (Table S1). BALF or sputum samples were collected from these patients.

### Microbe culture

BALF samples were sent to the Department of Clinical Laboratory for bacteria culture. Culture and strain identification were performed using a VITEK2 COMPACT automated ID/AST instrument (bioMérieux, France), as per the manufacturer’s instructions.

### DNA extraction and enzyme-based host depletion

BALF or sputum samples were divided into two parts (200 ul each) for standard DNA extraction and host DNA depletion. Standard DNA extraction was performed using the MagMAX CORE Nucleic Acid Purification kit per the manufacturer’s instructions.

Two types of methods exist for the enrichment of microorganisms in clinical samples. The first method is the direct capture of microbial sequences using primers or probes, but the target of these types of methods is limited. The second type is the reverse enrichment of microorganisms by removing the host nucleic acid. For example, a 5-um filter was used to remove intact human cells. The NEBNext kit enriches microorganisms by selective binding and removal of CpG-methylated host DNA. Microorganisms may also be enriched by the differential lysis of the host cells and removal of the released DNA. The differential lysis method was used to enrich pathogenic microorganisms in this study. Host DNA depletion and microbial DNA isolation were performed using a HostZERO Microbial DNA kit per the manufacturer’s instructions.

### Nanopore library preparation and sequencing

Approximately 1 μg of DNA extracted by the standard method was used to prepare a sequencing library using the Ligation Sequencing Kit (SQK-LSK109) according to the manufacturer’s instructions. For library cleanup, short fragment buffer (SFB) was used to retain DNA fragments of all sizes. The sequencing library for DNA extracted by the HostZERO Microbial DNA kit was prepared using the PCR Sequencing Kit (SQK-PSK004), according to the manufacturer’s instructions. Approximately 50 fmol of the prepared library was loaded onto the R9.4.1 flow cell. Sequencing was performed using the ONT GridION sequencing platform. Adaptive sequencing was applied using MinKNOW (21.10.6) software, which allowed us to deplete human sequences dynamically. Adaptive sequencing and standard sequencing for each library were performed simultaneously on the same flow cell by setting the adaptive sampling channel from 1 to 256 (leave 257–512 channels for standard sequencing). GRCh38 was used as the reference sequence. Host sequence depletion was enabled by selecting “Deplete”.

### Nanopore sequence analysis

The Fastq file was processed with porechop v0.2.4 (https://github.com/rrwick/Porechop) with the default parameters to trim off sequencing adapters. Trimmed reads were aligned to GRCh38 using minimap2 (2.17) with the parameters “-x map-ont -a -t 8” [29]. The preset option “-x map-ont” applies 15 as the minimizer k-mer length. The other minimap2 parameters were all set as default. As long as the read mapped to the GRCh38 reference, it was considered to be human, even when the value of mapping quality was 0, i.e., multiple mapped read. Human reads were set aside. The remaining unmapped reads were considered nonhuman reads. They were extracted from the sam format file using samtools with the command “samtools view -f 4” [30].

The nonhuman reads were aligned with our microorganism reference database using centrifuge (1.0.3) [31] with default parameters. The microorganism reference database was built by downloading Refseq-level sequences from the NCBI genome database (ftp://ftp.ncbi.nlm.nih.gov/genomes/). Sequences shorter than 150 bp were deleted. The final database was comprised of more than 20,000 reference genomes, including over 12,000 bacteria, 7312 viruses, 515 fungi, and 168 parasites. Reads aligned to multiple species were filtered out. Reads less than 100 bp in length were filtered out. Reads with sequences less than 50 bp aligned to the reference genome were also filtered out.

### Microbe identification

We developed a non-template-control (NTC)-based strategy to differentiate microbes from the experiment and reagent contamination. NTC was added for each batch of experiments, from DNA extraction to sequencing. After microorganism reference alignment and read filtering, standardized bases (bases per million sequencing bases (BPM), calculated as bases of a specific microbe/total sequencing bases × 1,000,000) were calculated for the sample and NTC. If a microbe was detected in the NTC, a BPM ratio (calculated as BPM-Sample/BPM-NTC) of 5 was used as the cut-off to differentiate the true positive microbes from the background. If a microbe was not detected in the NTC, a BPM value of 1 was used for reporting.

### Fastq normalization

Because the samples were sequenced on different flow cells, the number of reads obtained for each sample was not equal. To compare the removal of human reads and the microbial enrichment efficiency of the same sample by the four methods, we normalized the fastq data. Due to the uneven length of the sequenced reads, we normalized the four types of sequencing data for each sample according to the number of bases sequenced. Sequencing reads were randomly selected from each fastq file, until the total bases reach the standard number. The four fastq files sequenced from the same sample were processed with the same total base number.

### Statistical methods

We used a paired t-test in R (v4.1.0) to calculate the *p* value for the comparison of microbial reads, bases, number of genera and alpha diversity between the four methods. The number of microbial reads and bases were log10 transformed for p value statistics.

## Supplementary Information

**Additional file 1: Fig S1.** Study design.

**Additional file 2: Fig S2.** Nanopore sequencing read length of the four group, illustrated using P7 sample.

**Additional file 3: Fig S3.** Relative proportion of sequencing reads and bases of adaptive sequencing output. (A) SD_ADS group, (B) HD_ADS group.

**Additional file 4: Fig S4.** Read length of adaptive sequencing, illustrated using P4 sample. (A) SD_ADS group, (B) HD_ADS group. “unblock”: rejected reads, “stop receiving”: accepted reads, “no decision”: reads without decision.

**Additional file 5: Fig S5.** Mapping coverage of *E.coli* reads, illustrated using P1 sample processed with the SD_ADS method.

**Additional file 6: Table S1.** Patients enrolled in this study.

**Additional file 7: Table S2.** The number of reads and bases yielded by nanopore sequencing.

**Additional file 8: Table S3.** Relative proportion of human, *E.coli*, unclassified and microbe reads and bases in each sample.

**Additional file 9: Table S4.** Taxonomy classification results of the top 50 microorganisms in each sample obtained using normalized fastq files. For samples with the number of microbial species less than 50, all their microbial classification results were shown.

**Additional file 10: Table S5.** Taxonomy classification results of the top 50 microorganisms in each sample obtained using raw fastq files. For samples with the number of microbial species less than 50, all their microbial classification results were shown.

## Data Availability

The raw nanopore sequencing reads, after excluding human reads, have been deposited into BioProject (accession number PRJNA763343). The reference human genome used in this study was downloaded from NCBI (https://www.ncbi.nlm.nih.gov/assembly/GCF_000001405.26/).

## Abbreviations

SD_SSD: Standard DNA extraction with standard sequencing
SD_ADS: Standard DNA extraction with adaptive sequencing
HD_SSD: Host depletion with standard sequencing
HD_ADS: Host depletion with adaptive sequencing
BALF: Bronchoalveolar lavage fluid
HD: Host depletion
SD: Standard DNA extraction
ADS: Adaptive sequencing
NTC: Non-template-control
BPM: Bases per million sequencing bases
