# Supplementary figures and images for: Combined nanopore adaptive sequencing and enzyme-based host depletion efficiently enriched microbial sequences and identified missing respiratory pathogens
Source: BMC Genomics. 2021 Oct 9;22:732. doi: 10.1186/s12864-021-08023-0 (PMC8501638; doi:10.1186/s12864-021-08023-0)

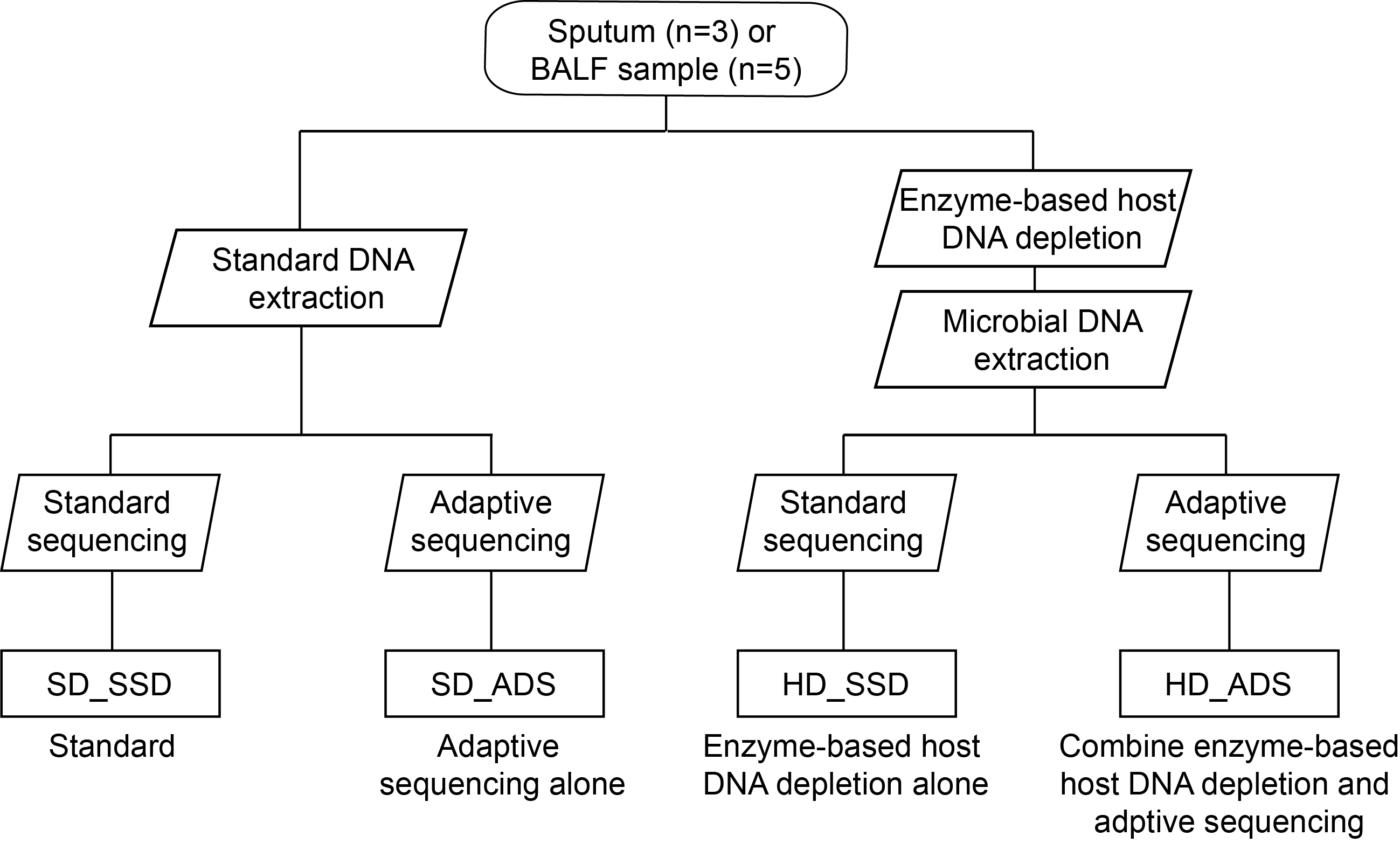

Supplement: Supplementary file 1 — Additional file 1: Fig S1. Study design. [file 12864_2021_8023_MOESM1_ESM.tif]

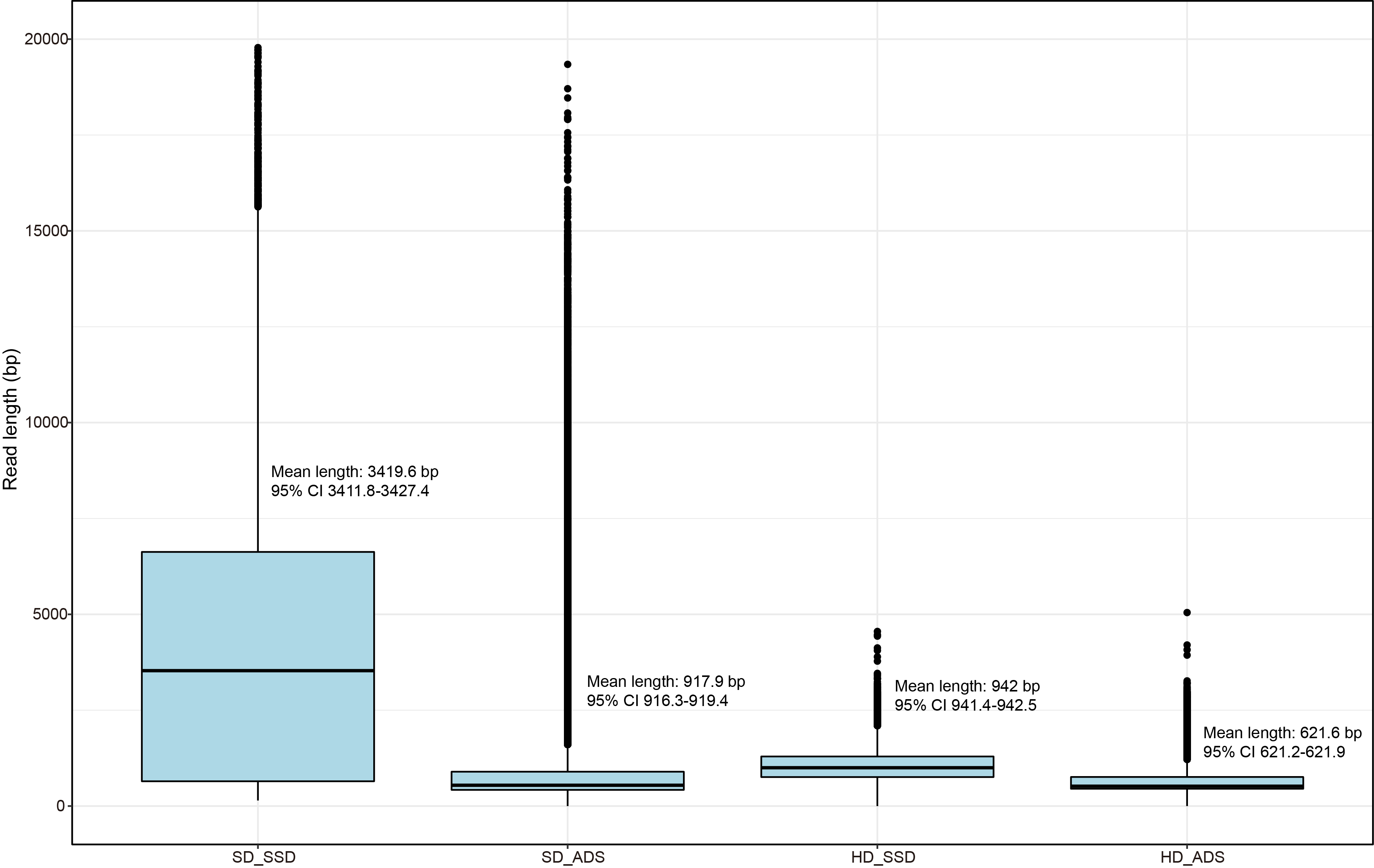

Supplement: Supplementary file 2 — Additional file 2: Fig S2. Nanopore sequencing read length of the four group, illustrated using P7 sample. [file 12864_2021_8023_MOESM2_ESM.tif]

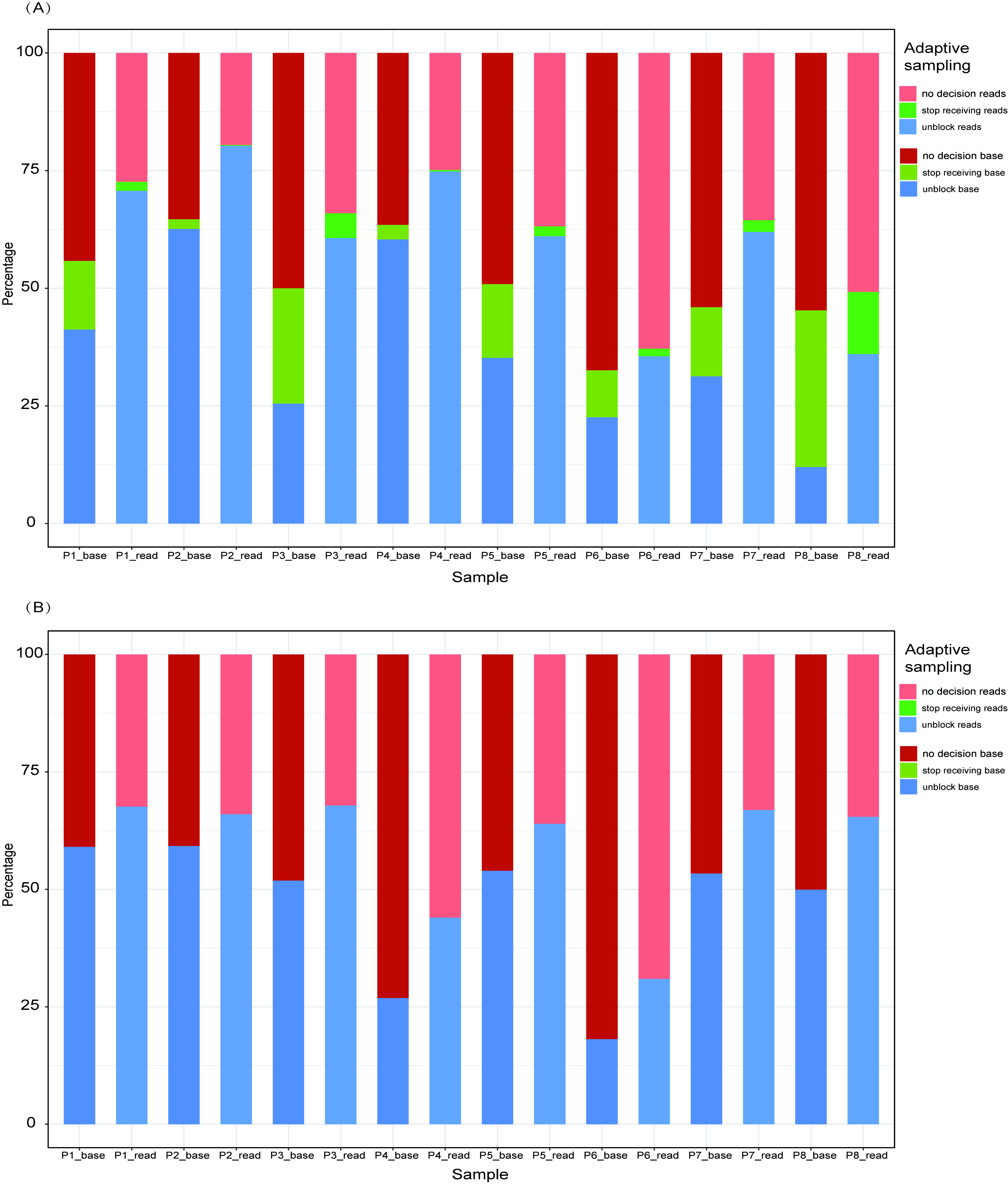

Supplement: Supplementary file 3 — Additional file 3: Fig S3. Relative proportion of sequencing reads and bases of adaptive sequencing output. (A) SD_ADS group, (B) HD_ADS group. [file 12864_2021_8023_MOESM3_ESM.tif]

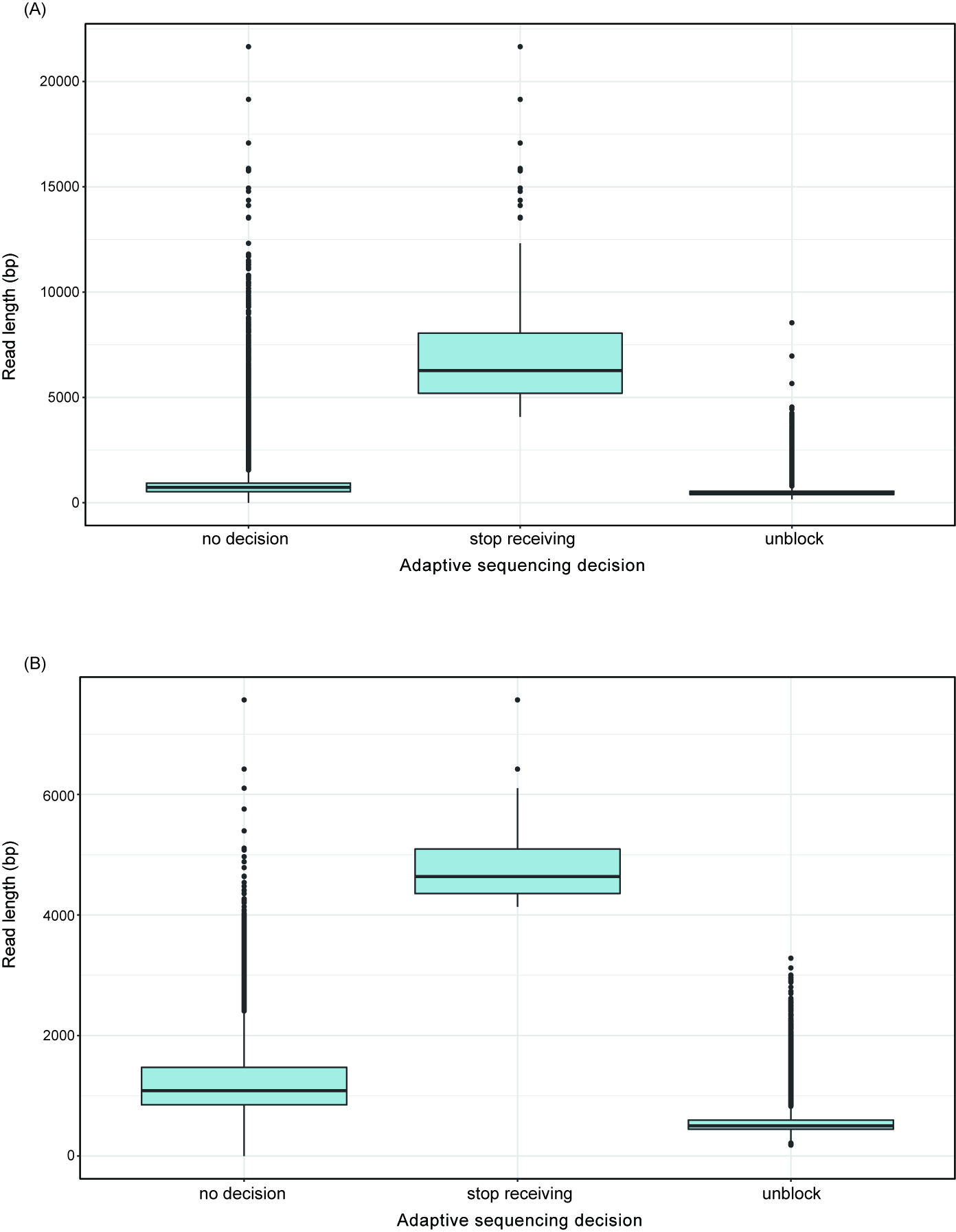

Supplement: Supplementary file 4 — Additional file 4: Fig S4. Read length of adaptive sequencing, illustrated using P4 sample. (A) SD_ADS group, (B) HD_ADS group. “unblock”: rejected reads, “stop receiving”: accepted reads, “no decision”: reads without decision. [file 12864_2021_8023_MOESM4_ESM.tif]

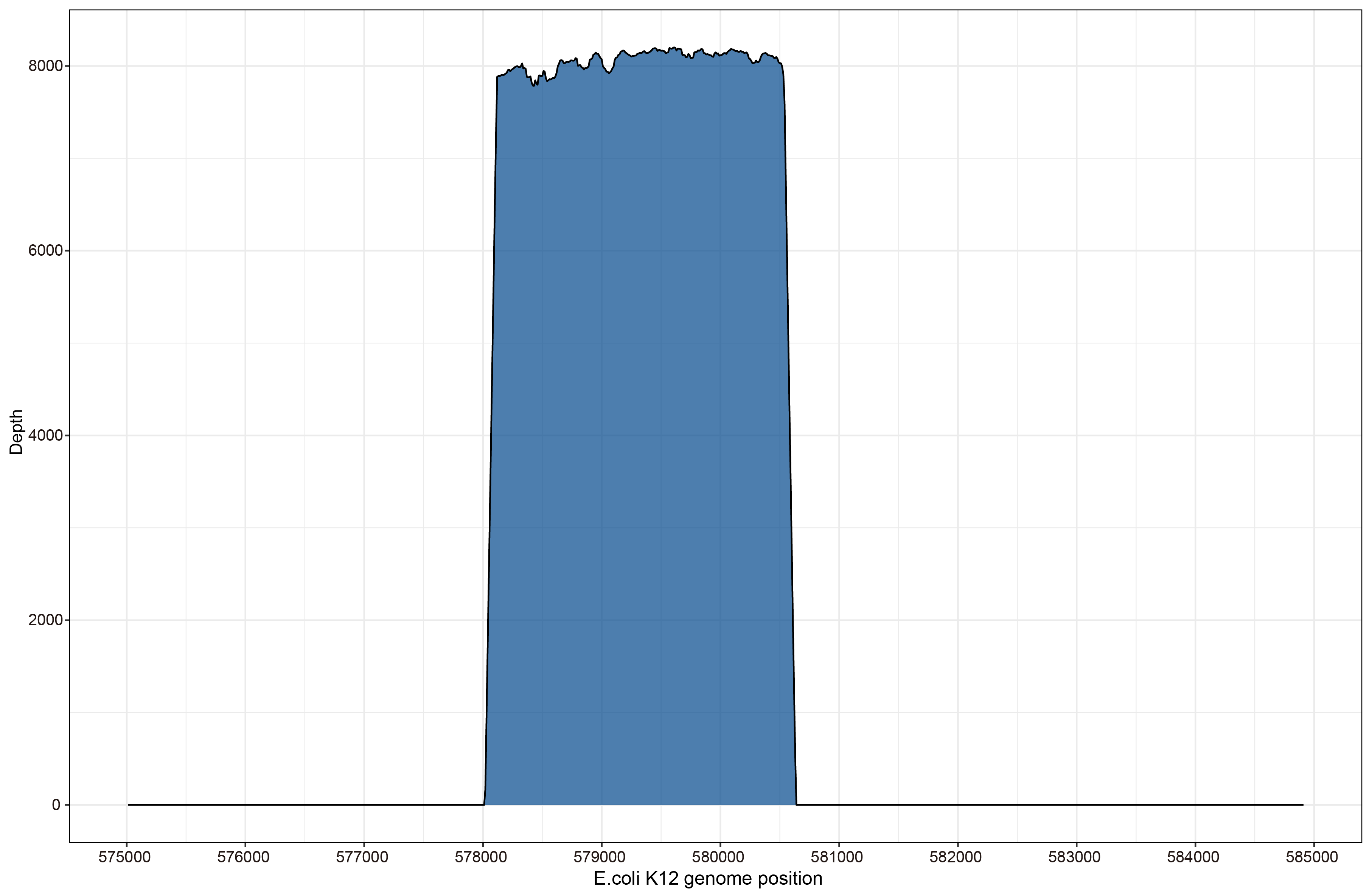

Supplement: Supplementary file 5 — Additional file 5: Fig S5. Mapping coverage of E.coli reads, illustrated using P1 sample processed with the SD_ADS method. [file 12864_2021_8023_MOESM5_ESM.tif]
